# Biliary Calculi

**Published:** 1868-08-01

**Authors:** E. W. Boyles

**Affiliations:** Clay City, Ill.


### BILIARY CALCULI.

BY E. W. BOYLES, M.D., OF CLAY CITY, ILL.

Called, June 6th, 1867, to see Mrs. W.; aged 50 years; nervo-bilious temperament. Found her suffering intense pain in right hypochondriac region; nausea and vomiting; pulse quick and feeble; bowels constipated; urine light colored. Upon examination found considerable enlargement and tenderness in right hypochondrium, extending into the umbilical and right lumbar regions. In fact, most all of the symptoms characteristic of acute hepatitis. Owing to the suddenness of the attack, and the paroxysmal character of the pain, I was led to suspect the presence of gallstones. Stated my opinion accordingly, and subsequently ordered the dejections examined therefor, but none were found. I gave opiates, and ordered fomentation. Called again next day and found my patient much relieved. She got up in a few days and attended her usual household duties, but tenderness and enlargement of the liver still remained to a considerable degree. I put her upon the use of nitro-muriatic acid—could not use mercurials, owing to the great susceptibility of the system thereto.

In September she was again confined to her bed for a few days with an attack similar to the first, though not so severe; after which she resumed her household duties. Treatment continued, together with various local remedies, such as iodine ointment, pustulation, blistering, etc.

January 4th, 1861, I was again called to see her. Tumor in the side larger, and more circumscribed. I became convinced that an abscess was forming, and used means to hasten the process of suppuration, fomentations and poultices, but the tumor remained hard—no fluctuation. I began to fear schirrhus; patient considerably emaciated and weak, but no appearance of jaundice. Digestion remarkably good, and plenty of bile in the stool.

The last of February, tumor began to point at the upper border of right lumbar, near the line of the umbilical region. March 1st, discharging slightly through two small sinuses about one and a half inches apart, which openings I enlarged with the lancet, after which discharged freely a fluid about the consistency and appearance of glycerine, which continued, producing great prostration; gave supporting remedies freely, in which iron predominated.

May 2nd, I was sent for again. Patient said to be suffering a great deal of pain; discharges from the abscess ceased, bulging between the openings — thought it must be lanced again. I was not at home at the time, and did not call until next day, when I found four gallstones had been discharged through the inferior opening, the first one being as large as a bird's egg, irregular in shape, weighing grs. xvij; the others about one-half the size and pyramidal in shape, with smooth, bright surfaces. Upon manipulation, four others were discharged whilst I was there. Others were discharged from day to day, until one hundred and six had come away, weighing, in the aggregate, two hundred and fifty grains. Most all of them pyramidal in shape, with smooth, bright surfaces. What seemed strange to me, there was no appearance of bile in the discharges from the abscess until May 20th, and then for a few hours only, and twice since that time, and at each there was more pain and gastric disturbance.

The upper opening has entirely closed, and the discharge from the other gradually growing less. The patient is rapidly improving, with every prospect of complete recovery; was at my house to-day (June 25th, 1868) visiting, having rode two miles in a spring wagon.

